# Prognosis Predictive Markers in Patients with Chronic Obstructive Pulmonary Disease and COVID-19

**DOI:** 10.3390/diagnostics13152597

**Published:** 2023-08-04

**Authors:** Nicoleta Ștefania Motoc, Iulia Făgărășan, Andrada Elena Urda-Cîmpean, Doina Adina Todea

**Affiliations:** 1 Department of Medical Sciences-Pulmonology, Faculty of Medicine, “Iuliu Haţieganu” University of Medicine and Pharmacy, 8 Victor Babeș Street, 400012 Cluj-Napoca, Romania; 2 Department of Medical Informatics and Biostatistics, Faculty of Medicine, “Iuliu Haţieganu” University of Medicine and Pharmacy, Louis Pasteur Str. No. 6, 400349 Cluj-Napoca, Romania

**Keywords:** COPD, COVID-19, SARS-CoV-2 infection, biomarkers, predictive model, mortality, intensive care unit, mechanical ventilation

## Abstract

Some studies have reported that chronic respiratory illnesses in patients with COVID-19 result in an increase in hospitalization and death rates, while other studies reported to the contrary. The present research aims to determine if a predictive model (developed by combing different clinical, imaging, or blood markers) could be established for patients with both chronic obstructive pulmonary disease (COPD) and COVID-19, in order to be able to foresee the outcomes of these patients. A prospective observational cohort of 165 patients with both diseases was analyzed in terms of clinical characteristics, blood tests, and chest computed tomography results. The beta-coefficients from the logistic regression were used to create a score based on the significant identified markers for poor outcomes (transfers to an intensive care unit (ICU) for mechanical ventilation, or death). The severity of COVID-19, renal failure, diabetes, smoking status (current or previous), the requirement for oxygen therapy upon admission, high lactate dehydrogenase (LDH) and C-reactive protein level (CRP readings), and low eosinophil and lymphocyte counts were all identified as being indicators of a poor prognosis. Higher mortality was linked to the occurrence of renal failure, the number of affected lobes, the need for oxygen therapy upon hospital admission, high LDH, and low lymphocyte levels. Patients had an 86.4% chance of dying if their mortality scores were −2.80 or lower, based on the predictive model. The factors that were linked to a poor prognosis in patients who had both COPD and COVID-19 were the same as those that were linked to a poor prognosis in patients who had only COVID-19.

## 1. Introduction

The coronavirus disease of 2019 (COVID-19) was declared a public health emergency in January 2020 and was officially categorized as a pandemic in March 2020 [1]. The presentation of COVID-19 was extremely heterogeneous, ranging from the absence of symptoms to severe, and sometimes fulminant, disease that was associated with high mortality [2,3]. Factors associated with poor outcomes, according to Center for Disease Control and Prevention, were age, cancer, cerebrovascular disease, preexisting conditions (such as chronic kidney disease, chronic respiratory disease, chronic liver disease, diabetes mellitus, heart conditions, HIV, mental health disorders, and neurologic conditions), obesity (body mass index (BMI) ≥ 30 kg/m^2^) and being overweight (BMI ranging from 25 to 29 kg/m^2^), physical inactivity, pregnancy or recent pregnancy, primary immunodeficiencies, smoking (current and former), and the use of corticosteroids or other immunosuppressive medications [4,5,6,7].

The prevalence of respiratory chronic diseases among COVID-19 patients varied greatly in various studies and according to the periods of time when such studies were published. In the initial reports, chronic respiratory conditions such as chronic obstructive pulmonary disease (COPD) and asthma had a lower prevalence in COVID-19 patients than in the general population [8], while in more recent studies, the prevalence was higher than it was in the general population [9,10]. The impact of respiratory chronic diseases on COVID-19 remains an intensely debated subject. While there were studies that showed poorer outcomes among patients with coexisting chronic respiratory disease, in terms of mortality and hospitalization, there were other studies that reported no significant difference in SARS-CoV-2 infection, hospital admission, or death between patients with chronic respiratory disease and patients without chronic respiratory disease [11,12]. In addition, some chronic respiratory conditions, such as asthma, were associated with a lower risk of death than other chronic respiratory conditions [13]. 

Some authors even suggested a certain protection from COVID-19 among asthma patients [14]. On the other hand, COPD has consistently been a risk factor for adverse outcomes of COVID-19. The high expression levels of ACE2 in the small airway epithelium of smokers and COPD patients have indicated that both COPD and smoking were indicators of a greater risk of adverse outcomes of COVID-19. In COVID-19 patients, the presence of COPD was associated with a greater probability of intensive-care-unit admission, mechanical ventilation, and death [15]. COPD prevalence in patients with severe acute respiratory syndrome coronavirus 2 (SARS-CoV-2) infection varied among the studies from 1.1% to 38%, based on the selection of patients and the time when the studies were performed. Such patients seemed to have a more severe form of COVID-19, a higher risk of death, a higher hospitalization rate, an increased chance of being admitted to an intensive care unit, and a higher likelihood of being mechanically ventilated [16]. As in the case of respiratory diseases and COVID-19, there were data supporting the contrary: i.e., that COPD does not have an impact on the development of a SARS-CoV-2 infection [17,18,19]. Considering the higher susceptibility of COPD patients to developing COVID-19, due to greater expression of ACE, and the reduced lung reserve usually found in such patients, the majority of the studies seemed to favor the first hypothesis: i.e., that COPD has a negative affect on the outcomes of COVID-19 patients. 

The primary objective of the current study was to determine markers (clinical, imaging, or blood tests) that could anticipate the outcomes in patients with COPD and COVID-19 As a secondary objective, we wanted to investigate whether a prediction model for poor outcomes could be created using such indicators. Such a model could help clinicians in their daily practice to quicky evaluate, based on certain markers at the time of hospital admission, a patient’s chances of a poor outcome, and then take the necessary measures to ensure the best possible recovery.

## 2. Materials and Methods

This prospective observational study took place in the “Leon Daniello” Clinical Hospital of Pulmonology in Cluj-Napoca (Romania), a first-line hospital in the battle against COVID-19.

Study population: In our study, 180 patients with a confirmed SARS-CoV-2 infection were included; they had previously been diagnosed with COPD and been consecutively admitted to the hospital from 27 March 2020 to September 2021. We used a patient sample of convenience, as we had access to only this hospital and we included all patients who had both diseases. COVID-19 diagnostics were confirmed using a real-time reverse-transcriptase polymerase-chain-reaction (RT-PCR) assay to test nasal and pharyngeal swab specimens, according to World Health Organization guidance. COPD had already been diagnosed by a clinician according to Global Initiative for Chronic Obstructive Lung Disease (GOLD) guidelines. In Romania, hospitalization was compulsory for all patients diagnosed with COVID-19, regardless of the disease’s clinical severity. Therefore, the patients had a mild, moderate, or severe form of COVID-19. Inclusion criteria consisted of all hospitalized patients with confirmed COVID-19 and COPD who gave their consent to participate in the study. Exclusion criteria included patients with confirmed COVID-19 and cancer, hematological diseases, severe cardiac disease (NYHA III and IV cardiac failure, recent myocardial infarction in the last three months, unstable arrhythmia), liver disease, systemic diseases, or pulmonary fibrosis. In addition, patients who did not wish to not participate and patients with missing data were excluded.

Study design: We collected patients’ demographic, clinical, laboratory, and treatment data (if available) at hospital admission. If such data were not available, we extracted the data from the hospital’s electronic medical records. At the time of hospital entry (i.e., before any intervention), we collected samples from all patients for laboratory tests and recorded the test results. 

Blood examinations involved measuring complete blood cell counts and differential values. Serum bio-chemical tests were carried out, and erythrocyte sedimentation rates, C-reactive protein levels, procalcitonin levels, D-dimer levels, and serum ferritin levels were determined for the COVID-19 patients. All laboratory tests were carried out in the hospital laboratory with standard procedures. The laboratory reference values for white blood cells, neutrophils, lymphocytes, and eosinophils were 4.2–10, 1.8–7.3, 1.5–4, and 0.05–0.35 × 10^3^/μL, respectively. For ferritin, the values were 30–220 μg/la, and D-dimers were considered positive if they were above 500 ng/mL FEU (25–5000 ng/mL FEU). For procalcitonin, a value above 0.5 ng/mL was considered suggestive of bacterial infection.

The NLR ratio was calculated as the absolute count of neutrophils divided by the total count of lymphocytes. The PLR ratio was defined as: the absolute count of platelets divided by the absolute count of lymphocytes. 

We also collected the following: clinical markers (age, number of days from the onset of symptoms until hospitalization, number of hospitalization days, smoking status, BMI, previous medication, previous oxygen therapy, and type of oxygen therapy administered at admission), imaging markers (severity of lung involvement, the presence of consolidation), and paraclinical parameters (blood tests). We assessed COVID-19 disease severity in all cases, using the following criteria [20]:

- mild disease: mild symptoms without dyspnea or pneumonia;
- moderate disease: evidence of lower respiratory disease by clinical assessment or imaging and a saturation of oxygen (SaO_2_) ≥ 94 percent in room air at sea level;
- severe disease: tachypnea (respiratory rate > 30 breaths/minute), hypoxia (oxygen saturation ≤93% in room air or PaO_2_/FiO_2_ < 300 mmHg), or >50% lung involvement on imaging;
- critical care: involving respiratory failure, shock, or multiorgan dysfunction.

The types of oxygen therapy administered at admission were No O_2_ (no mask), nasal canula (NC), simple O_2_ mask (SM), ventury mask (VM), non-rebreathable mask (NRM), and high flow oxigen therapy(HFOT).

Regardless of disease severity, we performed chest computer tomographies (CTs) in the hospital radiology department for all the patients. Subsequently, the images were reviewed by a radiologist and a pulmonologist (the same radiologist and the same pulmonologist were involved throughout the entire study). Typical imaging findings in COVID-19 patients included ground-glass opacities (GGO) with peripheral and subpleural distribution, usually involving lower lobes. The CT severity score used in our study was the total severity score (TSS) proposed by Li et al. It has five grades of severity of involvement for five lung lobes: 0%, 1–25%, 26–50%, 51–75%, and 75–100%. The higher the score, the more severe the lung involvement [21]. Consolidation on thoracic CT in COVID-19 patients might be a sign of bacterial infection; therefore, those situations were noted separately. 

Poor outcomes were defined as a patient’s transfer to the intensive care unit (ICU) for mechanical ventilation (invasive or noninvasive) and death of a patient. 

A good outcome was defined as discharge of a patient who was either completely healed or whose health had improved.

Statistical analysis was performed using the IBM (Richmond, VA, USA) SPSS STATISTICS 25 application. Qualitative data were presented using frequencies and percentages. The Kolmogorov–Smirnov test was used to test data distribution. Median values (25th percentile to 75th percentile) were calculated for quantitative variables with a non-normal distribution; means and standard deviations were calculated for quantitative variables with a normal distribution. The comparison of independent samples was tested with the Kruskal–Wallis test for non-normally distributed data. Frequencies were compared with the Chi-square test or the Fisher exact test. The significance level was set at *p* < 0.05. The event considered was the recovery of the patient (a good outcome), which was the opposite of the poor outcomes (transfer to the intensive care unit for mechanical ventilation or death). We used this variable as dependent in a multivariate logistic regression. All significant variables from the univariate analysis were introduced into the entered model as independent variables. A *p*-value < 0.05 was regarded as statistically significant. A logistic regression was performed to develop a model that might help in predicting outcomes in these patients. To evaluate the correctness of the fit of the logistic regression model, the Nagelkerke’s R-squared value was computed (the model’s power of explanation). For each possible model, a formula with specific biomarkers was presented in detail. The area under the curve (AUC) with a 95% confidence interval and the score cutoff points were calculated, based on the patients’ score result and on the poor/good outcomes. 

## 3. Results

Among the patients hospitalized with COVID-19 in the hospital, COPD prevalence was 7% (180 out of 2570 patients). All relevant data was available for only 165 patients. The patients’ demographics characteristics are shown in Table 1. Most patients were male and over 65 years old, with a cardiovascular disease. 

### 3.1. Markers (Clinical, Imaging, or Blood Tests) That Could Predict the Outcomes in Patients with Both COPD and COVID-19

#### 3.1.1. Risk Factors for Non-Invasive Ventilation Prognosis

To identify the significant risk factors, univariate logistic regression was used and one factor at a time was introduced into the modeling (Table 2 and Table 3). The dependent variable was the binary variable for non- invasive ventilation (Yes/No).

The logistic regression included COVID-19 severity, consolidation, number of affected lung lobes, eosinophiles, PCR, LDH, neutrophiles, lymphocytes, leucocytes, thrombocytes, diabetes, renal failure, respiratory failure, pre-existing treatment present, O_2_-therapy type at admission, smoker status, and non-invasive ventilations. 

In this predictive model, 61.7% (Nagelkerke’s R-squared value) of the variance of non-invasive ventilation were explained by the number of affected pulmonary lobes (1 lobe), high PCR values, and the presence of diabetes. 

Based on these significant markers, a score was computed for each patient, using the beta-coefficients from the logistic regression model:

Non-invasive ventilation score =

if 1 lobe was affected, then −3.39, otherwise 0

+ PCR value * 0.02

+ if diabetes present, then 1.15, otherwise 0

− 6.2 (constant).

**Figure 1:**
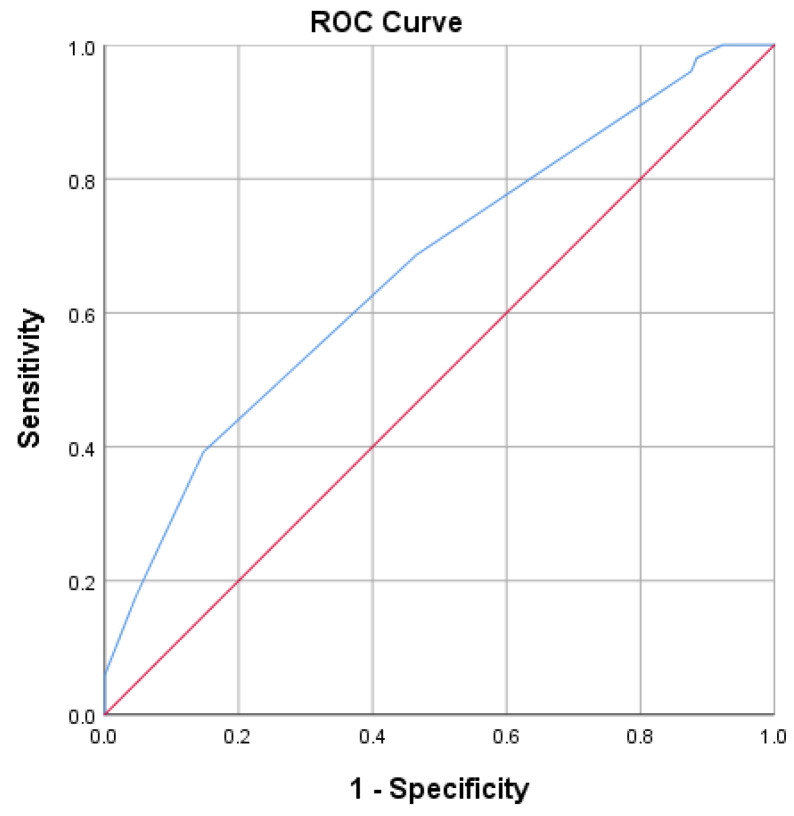
ROC curve (blue line) for predicting non-invasive ventilation using the non-invasive ventilation score.

The area under the curve (AUC) for the non-invasive ventilation score and non-invasive ventilation was 66.7% (95% CI [57.8–75.6%], *p* < 0.001) (Figure 1).

A patient with a non-invasive ventilation score of −4.5 or lower had an 85% chance of being placed on non- invasive ventilation.

#### 3.1.2. Risk Factors for ICU and Invasive Mechanical Ventilation Prognosis

To identify the significant risk factors, univariate logistic regression was used and one factor at a time was introduced into the modeling (Table 4 and Table 5). The dependent variable was the binary variable for ICU and invasive mechanical ventilation (Yes/No).

The logistic regression included COVID-19 severity, consolidation, number of affected lung lobes, eosinophiles, PCR, LDH, neutrophiles, lymphocytes, leucocytes, thrombocytes, renal failure, diabetes, O_2_ therapy type at admission, smoker status, non-invasive ventilation, and pre-existing treatment. 

In this predictive model, 78% (Nagelkerke’s R-squared value) of the variance of invasive mechanical ventilation in the ICU was explained by the number of affected pulmonary lobes (two lobes), active smoker status, pre-existing pulmonary treatment, and existing non-invasive ventilation. 

Based on these significant markers, a score was computed for each patient, using the beta-coefficients from the logistic regression model:

Invasive ventilation score =

if two lobes were affected, then −14, otherwise 0

+ if active smoker, then −13.4, otherwise 0

+ if with non-invasive ventilation, then +8, otherwise 0

+ if with existing pulmonary medication, then −8.3, otherwise 0.

**Figure 2:**
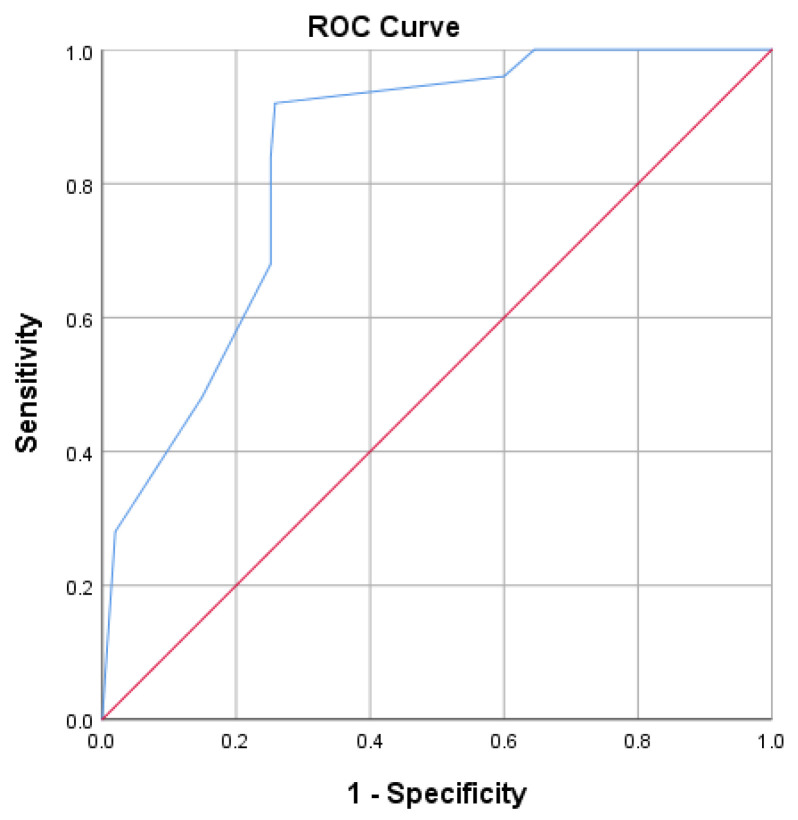
ROC curve for predicting ICU and invasive mechanical ventilation using the invasive ventilation score.

The area under the curve (AUC) for the invasive ventilation score and invasive mechanical ventilation in the ICU and was 83.8% (95% CI [76.8–90.8%], *p* < 0.001) (Figure 2).

A patient with an invasive ventilation score of −7.15 or lower had a 74.2% chance of being transferred to the ICU and being placed on invasive mechanical ventilation.

#### 3.1.3. Risk Factors for Death Prognosis

To identify the significant risk factors for a prognosis of death, univariate logistic regression was used one factor at a time and introduced into the modeling (Table 6 and Table 7). The dependent variable was the binary variable of death (Yes/No).

The logistic regression included renal failure, non-invasive ventilation, invasive mechanical ventilation in the ICU, lymphocytes, and pre-existing treatment.

In this predictive model, only 38.7% (Nagelkerke’s R-squared value) of the variance of death were explained by the presence of renal failure and existing mechanical ventilation in the ICU. 

Based on these significant markers, a score was computed for each patient, using the beta-coefficients from the logistic regression model:

Mortality Score =

if renal failure present, then 1.36, otherwise 0

+ if with mechanical ventilation in the ICU, then +3.75, otherwise 0

− 3.48 (constant).

**Figure 3:**
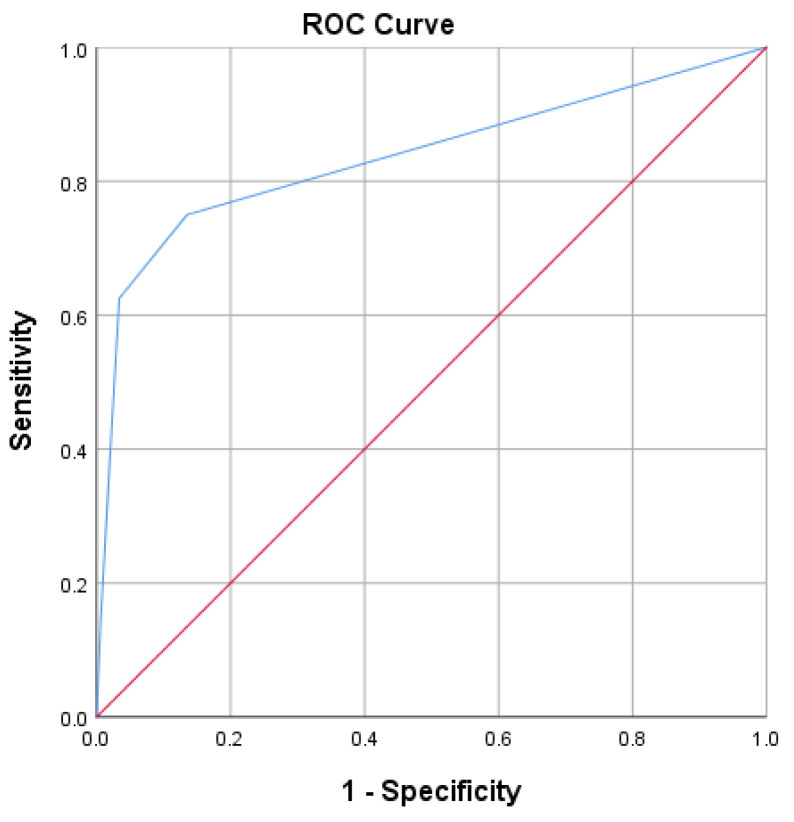
ROC curve for predicting death, using the mortality score.

The area under the curve (AUC) for mortality score and death was 83.8% (95% CI [74.3–93.2%], *p* < 0.001) (Figure 3).

A patient with a mortality score of −2.80 or lower had an 86.4% chance of dying.

For example, a male patient with declining health was initially prescribed non-invasive ventilation, then invasive mechanical ventilation, but then he died. He had three affected lobes, a PCR = 8.8, no renal failure, an existing pulmonary medication, was a non-smoker, and had diabetes, with the following scores:

- Non-invasive ventilation score = −4.87 (power of predictive model 61.7%, chance of non-invasive ventilation), which was lower than −4.5; thus, he had an 85% chance of being placed on non- invasive ventilation.
- Invasive ventilation score = −0.3 (power of predictive model 78%, chance of invasive mechanical ventilation), which was greater than −7.15, and he had a 74.2% chance of being transferred to the ICU and being placed on invasive ventilation.
- Mortality score = 0.27, which was higher than −2.80. Since there were only 25 deaths in the sample, this model had low precision and only 38.7% of the variance of death was explained by the above markers.

### 3.2. Good versus Poor Prognosis

Non-invasive ventilation was prescribed to 25.45% (42/165) of the hospitalized patients, but for 30.1% (13/42) of those non-invasive ventilated patients needed to be transferred to the ICU for mechanical ventilation (invasive) and, in the end, 9 of those 13 patients died (Table 8).

## 4. Discussion

The present study analyzed prognosis predictive markers in patients hospitalized with concomitant COPD and SARS-CoV-2 infection. Out of 165 patients with complete data, 55 had a poor prognosis (non-invasive/invasive mechanically ventilation or death), and 110 patients survived with no ventilation at all. We used a sample of all patients from the beginning of the pandemic in March 2020 until September 2021, during which period COVID-19 patients were hospitalized in Romania, regardless of the COVID-19 severity. Therefore, the sample was varied and random. 

The negative prognostic factors identified in the present research were COVID-19 severity, the presence of renal failure, the presence of diabetes, smoking status (active or former), the requirement of oxygen therapy at admission, high values of LDH and CRP, and low values of eosinophils and lymphocytes. The presence of renal failure, the number of affected lobes, the requirements of oxygen therapy at admission, high LDH, and low lymphocytes values were associated with higher mortality. Based on the significant discovered markers for each defined prognosis (non-invasive ventilation, invasive ventilation, or death), a score was computed using the beta-coefficients from the logistic regression.

Although the predictive models were all statistically significant (*p* < 0.05), the power of each prediction model to explain the model differed. Of the three prediction models using biomarkers, the invasive ventilation score description was the most accurate (78%). Furthermore, its AUC of 83.8% (95% CI [76.8–90.8%]) demonstrated a moderate-to-good prediction of patients being provided with invasive mechanical ventilation in the ICU.

A non-invasive ventilation score of −4.5 or lower indicated an 85% likelihood of a patient being placed on non-invasive ventilation. A patient’s risk of being admitted to the ICU and receiving invasive mechanical ventilation was 74.2% if their invasive ventilation score was −7.15 or below. A patient had an 86.4% probability of dying if the patient’s mortality score was −2.80 or lower. Models for predicting non-invasive and invasive ventilation scores took into account the number of affected lobes, which reflected the severity of a SARS-CoV-2 infection. The most important parameter for the mortality score, as expected, was invasive mechanical ventilation. Based on the results, we assumed that the prognosis of patients with both COPD and COVID-19 depended mostly on the severity of COVID-19 rather than on COPD status. Since our study did not address the evaluation of COPD severity—a study limitation—we were unable to provide any definitive conclusions. 

The identified poor-prognosis risk factors in our study were the same as the risk factors for poor prognosis in COVID-19 cases: i.e., certain comorbidities (renal failure and diabetes), active or former smoker status, and the extension of pulmonary lesions [4,5,6]. Our findings were consistent with those that suggested that COPD had no impact on the outcomes for COVID-19 patients [17,18,19]. As patients with pre-existing chronic diseases may be more vulnerable to organ failure, as a result of their altered previous status, this factor may encourage the development of more severe types of COVID-19. The prevalence of COPD in our study was 7%. According to a study carried out by the Romanian Society of Pulmonology in 2019, the prevalence of COPD in the general population was 8.3% in patients over 40 years old [22]. 

Thus, COPD was more prevalent in the general population than it was among COVID-19 patients. In a retrospective cohort, the prevalence of respiratory disease among COVID-19 patients at the beginning of pandemic was very low—1.6% for chronic obstructive pulmonary disease (COPD) and 0.6% for asthma—lower even than the prevalence of such respiratory diseases in the general population (8.6% and 4.2%, respectively) [8]. The low prevalence was confirmed by other studies [23,24,25]. This was in contrast with the UK study of the International Severe Acute Respiratory and Emerging Infection Consortium (ISARIC), World Health Organization Clinical Characterization Protocol (UK study), which reported a higher proportion of patients with asthma (10.4%) and non-asthmatic chronic pulmonary diseases (18%) among patients with COVID-19, as compared with the general population [9].

There are, however, a few factors that must be taken into consideration when considering the differences among these observational studies. First, there could have been some under-reporting of data, as the first studies focused on hospitalized and intensive-care-unit (ICU) patients, rather than on mild outpatient cases. Second, the means used by clinicians to collect data in the beginning of the pandemic, when everything was new and unknown, plus the lack of a nationwide standardized electronic data registry system in all countries, contributed to these differences. Finally, there was an underdiagnosis of COPD in many countries (including Romania) during the pandemic, as spirometry was not performed, to avoid contamination. 

The patients included in the present study had stage 2 COPD (a moderate form) and most of them (71.52%) were receiving inhaled treatment. Compliance with the treatment was, unfortunately, not determined. This could explain why previous medication administration, as a parameter, was associated with poor prognosis in patients who were invasively ventilated. Another explanation could be that those patients had more severe or symptomatic forms of COPD. We did not find any relationship between GOLD-stage COPD and outcomes; it would have been useful to determine the outcome by including a consideration of COPD severity. Most of our patients were typical of the general population with respect to COPD and COVID-19: i.e., male, over 65 years old, and former-or-active smokers. Age and sex are known prognostic factors for many chronic diseases. For COVID-19, age was a prognostic factor related to hospitalization and mortality, presenting a linear dose–response association with mortality. Although large between-study heterogeneity was observed, in most studies, the risk of severe disease rose steadily with age, with more than 93% of deaths occurring among adults ≥ 50 years and 74% of deaths occurring in adults ≥65 years [6]. Sex was identified as a prognostic factor for ICU admission, acute kidney injury, invasive mechanical ventilation, and a composite outcome (defined as ICU admission and death). In these situations, small or moderate between-study heterogeneity was observed, but 95% of the prediction intervals included a null value for acute kidney injury and a composite outcome. We did not find any relationship with COPD severity, as assessed by lung function. In a recent study published by Yeung et al., which looked into the association of smokers’ lung function and COPD in COVID-19, the authors did not find any relationship between lung function and COPD, while they found that smoking increased the risk of COVID-19, compared with population controls, for overall COVID-19, including the life-time smoking index odds ratio (OR) of 1.19 with a 95% confidence interval (CI) [1.11–1.27], hospitalization with COVID-19 (OR  =  1.67, 95% CI  [1.42–1.97]), and severe COVID-19 (OR  =  1.48, 95% CI  [1.10–1.98]), with directionally consistent effects based on sensitivity analyses [26].

In another study, smoking was an important risk factor for poor outcomes in COVID-19 patients. SARS-CoV-2 bears an envelope spike protein that is primed by the cellular serine protease TMPRSS2 to facilitate fusion of the virus with the cell’s angiotensin-converting enzyme 2 (ACE-2) receptor and subsequent cell entry [27,28]. ACE-2 expression was significantly elevated in COPD patients, compared to control subjects. Current smoking was also associated with higher ACE-2 expression, compared with that of former smokers or patients who had never smoked, an observation that has subsequently been validated by other groups in separate reports on lung tissue and airway epithelial samples and supported by additional evidence linking ACE-2 expression to nicotine exposure [27,29].

Nonetheless, there was increasing evidence that COPD may be a risk factor for more severe COVID-19 disease. An analysis of comorbidities in 1590 COVID-19 patients found that COPD carried an odds ratio of 2.681 (95% CI [1.424–5.048]; *p* = 0.002) for ICU admission, mechanical ventilation, or death, even after adjustment for age and smoking; 62.5% of the severe cases had a history of COPD (compared with only 15.3% of the non-severe cases), and 25% of those patients who died were COPD patients (compared with only 2.8% of those who survived). In a multicenter study, COPD patients made up 15.7% of the critically ill patients, but only 2.3% of the moderately ill patients (*p* < 0.001) [15]. Other studies found similar, but statistically weaker, differences in COPD rates between ICU-admission patients and non-ICU-admission patients (8.3% versus 1.0%; *p* = 0.054) [9], between severe and non-severe cases (4.8% versus 1.4%; *p* = 0.026) [16]; and between non-survivors and survivors (7% versus 1%; *p* = 0.047). The same conclusion was drawn from the research performed by Lee et al., in which they concluded that even though COPD was not a risk factor for respiratory failure, it was a significant independent risk factor for all-cause mortality (OR  =  1.80, 95% CI [1.11–2.93]) [30]. Among COVID-19 patients, relatively greater proportions of patients with COPD received mechanical ventilation and intensive critical care [16,30,31].

Acute severe respiratory failure associated with COVID-19 was characterized by severe hypoxemia, with good lung compliance [16]. Similar risk factors have been described by Bellou et al. in a recent systematic review that evaluated, in over 400 articles (observational studies, meta-analyses), prognostic factors for adverse outcomes in patients with COVID-19. The following parameters were associated with a poor outcome: age, sex, smoking status, the presence of dyspnea, oxygen saturation at admission, obstructive sleep apnea, venous thromboembolism, cardiovascular disease, chronic kidney disease, chronic lung disease, diabetes mellitus, obesity, cancer, chronic liver disease, COPD, dementia, peripheral arterial disease, and rheumatological disease [32].

We decided to mark the consolidation cases separately, as those cases could be a sign of bacterial infection. Consolidation is defined as a homogenous high density (area of increased attenuation) that obscures the bronchial and vascular markings (airway walls and blood vessels). It is caused by the filling of the alveolar spaces with fluid, exudates, transudate, blood, or neoplastic cells. In this condition, alveolar air is replaced by other materials (e.g., pathological fluids, cells, or tissues), with a subsequent increase in pulmonary parenchymal density. It has bilateral, multifocal, and subsegmental distribution. The presence of consolidation is considered a sign of progressive COVID-19 disease, as it develops in the second week after the onset of symptoms. It is seen more in patients who are over 50 years old; therefore, consolidation could serve as a clue for an illness that necessitates greater vigilance in management [33].

According to another review, consolidation in COVID-19 pneumonia tended to be patchy or segmental, irregular, or nodular, and mainly subpleural and peripheral, with a reported incidence of 2–64%, depending on the duration of the illness. It usually appears 10 to 12 days after the onset of symptoms—after the appearance of GGO. One study reported high mortality in patients with consolidation. Another study of 83 patients reported consolidation in patients with severe or advanced disease. In a different study, the incidence of consolidation was significantly higher in older patients (>50 years) and significantly higher in patients who had symptoms for more than 4 days [34].

Air bronchogram, which is defined as air-filled bronchi in areas with high density, has variable incidence in different reports, ranging from 28% to 80% of patients. It is usually a sign of advanced disease and is usually seen after the second week from the onset of symptoms. It can be seen in both GGO and consolidation cases [21,34].

As COVID-19 and bacterial pneumonia are different clinical entities from COPD acute exacerbation (although they might/frequently coexist), we decided to describe the consolidation as if they are very frequent, as they might suggest that COPD increases the risk of bacterial complication in patients with SARS-CoV-2 infection. Bacterial infection was not very common in COVID-19 patients, especially not at the beginning of the disease. Since it could appear later, as a complication of the disease or because of medical care (especially invasive medical care), it is worth reflecting on the innate altered immune response that is involved in severe forms of COVID-19 [34]. 

If bacterial pneumonia could coexist with COPD, why would COVID-19 not coexist—especially if we consider SARS-CoV-2 a virus that could be a trigger for exacerbation? 

An acute exacerbation of COPD represents the aggravation of respiratory symptoms beyond day-to-day variation that requires a change in medication. Eighty percent of the acute exacerbations were linked either to bacterial or viral pathogens. More than half of them had a bacterial etiology [35,36]. The most frequent germs that have been isolated during COPD exacerbation were streptococcus pneumoniae, hemophilus influenza and moraxella catharalis, but we must consider that microorganisms are commonly detected in the airways in stable COPD cases and are considered to be “colonizers” in the absence of acute infective symptoms [37].

The term “colonization” is debatable, as the microorganisms identified in stable COPD cases are not necessarily benign. As a subset of COPD patients has frequent exacerbations, the concept of the inherent susceptibility to acute infection in COPD cases subsequently triggering AECOPD events has been developed. The impaired innate immune response favored by smoking results in bacterial colonization, which promotes airway and systemic inflammation, leading to COPD progression and exacerbation. To this, we add the antibiotic-mediated lung dysbiosis during therapy [37]. Defects in innate immunity could also play a role in increased susceptibility to viruses. Recognition of viral infection by the innate immune system is essential for coordinating an effective antiviral response in the airways; however, in patients with COPD, the cascade from recognition to response falters, due to exposure to cigarette smoke that diminishes the antiviral response [38]. Furthermore, mucociliary clearance, which is key for the removal of virus from the airways, appears to be perturbed in COPD, as cigarette smoking exposure reduces both the number and the length of cilia, while goblet cell hyperplasia in COPD leads to more viscous mucus in the airways, further impeding proper ciliary motion [38,39,40].

Since the SARS-CoV-2 virus shares common pathobiological and clinical features with other viral agents, it could trigger COPD exacerbation, with the potential for a more long-term adverse impact. Nevertheless, COVID-19 and AECOPD are different clinical entities, although they could coexist, making it very difficult to differentiate the two. When an exacerbation of COPD occurred during COVID-19, the usual guidelines called for initiation of systemic glucocorticoids, as recommend by the GOLD guidelines. For patients hospitalized with COVID-19, the use of nebulized medications should be avoided or limited to negative pressure rooms because of the risk of aerosolizing SARS-CoV-2 and enhancing the spread of disease. Clinical outcomes, including mortality, are worse in males, older individuals, and patients with comorbidities. COPD patients are included in shielding strategies because of their susceptibility to virus-induced exacerbations, compromised pulmonary function, and a high prevalence of associated comorbidities [39]. Most of our patients had corticosteroids (ICS) in their treatment, in a fixed combination with a bronchodilator. In case of diagnostic uncertainty, we advise physicians to be careful about initiating ICS or ICS/long-acting β-agonist in patients in the absence of clear objective evidence of asthma. Similarly, there was no evidence to suggest a change in the advice that the dose of ICS for asthma patients be increased at the onset of exacerbation [16].

However, we believe that our findings are pertinent, as they demonstrate the significance of COVID-19 severity in patients’ outcomes for chronic respiratory disorders. A medical team should act more quickly and take more drastic measures as a result of the presence of observed poor prognosis variables in patients with COPD and COVID-19. We believe that it is crucial to be ready to provide better care of these patients by knowing that the prognosis is impacted by the infection and not by the respiratory disease, as the virus is here to stay and its capacity to adapt is rather impressive [41].

Limitations: This was a single-center study; therefore, certain aspects cannot be generalized. Second, the sample had mostly good outcomes, but for better prognostic models, more cases with poor outcome are needed. Third, COPD diagnosis could have been underestimated, given that spirometry was not performed during the pandemic period and the disease might have been even more prevalent. The COPD risk class was not assessed; consequently, COPD severity could have an impact on COVID-19 impact and not be detected in our study. We did not assess the relationship between COPD severity and patients’ outcomes. This was important, as in Romania all patients with COVID-19 were hospitalized regardless of the COVID-19 severity, so the population was very heterogenous. 

## 5. Conclusions

The factors identified in the current research that were linked to a poor prognosis in patients with COPD and COVID-19 were similar to those linked to a poor prognosis in patients with COVID-19 alone. The severity of COVID-19 affected patient outcomes far more than COPD itself. Although COPD patients may be more at risk for COVID-19 infection, COVID-19 seemed to have an influence on how the disease progressed.

## Figures and Tables

**Table 1:** Distribution of demographic characteristics and comorbidities for patients with COVID-19 and COPD.

Factors *n* = 165	*n* (%)	Factors *n* = 165	*n* (%)
Location Rural Urban	82 (49.7) 83 (50.3)	Smoking status Active smoker Former smoker Never-smoker data	40 (24.3) 42 (25.4) 83 (50.3)
Gender Masculine Feminine	127 (77) 38 (23)	Comorbidities present *n*(%) Cardiovascular disease Arterial hypertension Diabetes Renal failure Respiratory failure Treatment	136 (82.4) 136 (82.4) 61 (36.9) 22 (13.3) 55 (33.3) 118 (71.52)
Age <65 years ≥65 years	42 (25.45) 123 (74.5)		
**Spirometry parameters**	**m ± SD**	**50% (25–75%)**	
FVC%	73.98 ± 22.68	72.4 (61.9–89.1)	
FEV1%	66.89 ± 26.49	63.9 (50.15–83.58)	
FEV1 (L)	1.78 ± 0.53	1.77 (1.42–2.07)	
MEF 50	43.71 ± 30.99	36.2 (19.73–60.23)	

**Table 2:** Qualitative markers for non-invasive ventilation.

		Non-Invasive Ventilation		
Qualitative Markers		Present (*n* = 42) *n* (%)	Absent (*n* = 123) *n* (%)	*p*-Value
COVID-19 severity	Severe	39 (92.9%)	79 (64.2%)	**0.025 ^a^**
	moderate	2 (4.8%)	25 (20.3%)	
	light	1 (2.4%)	19 (15.4%)	
Consolidation		28 (66.7%)	51 (41.5%)	**0.005 ^a^**
Number of affected lobes = 0		17 (40.5%)	76 (61.8%)	**<0.001 ^a^**
Cardiovascular disease present		34 (81%)	102 (82.9%)	0.468 ^b^
Arterial hypertension present		37 (88.1%)	99 (80.5%)	0.263 ^a^
Diabetes present		22 (52.4%)	39 (31.7%)	**0.017 ^a^**
Renal failure present		10 (23.8%)	12 (9.8%)	**0.021 ^a^**
Respiratory failure present		16 (38.1%)	39 (31.7%)	0.448 ^a^
Pre-existing treatment present		31 (73.8%)	87 (70.7%)	0.703 ^a^
O_2_-therapy type at admission	No O_2_	3 (7.2%)	21 (17.1%)	**<0.01 ^a^**
	NC	0 (0%)	32 (26.1%)	
	SM	3 (7.2%)	19 (15.5%)	
	VM	8 (19.1%)	25 (20.4%)	
	NRM	25 (59.6%)	24 (19.6%)	
	HFOT	3 (7.2%)	2 (1.7%)	
Smoker status	Non-smoker	27 (64.3%)	56 (45.5%)	**0.044 ^a^**
	Former smoker	5 (11.9%)	37 (30.1%)	
	Active smoker	10 (23.8%)	30 (24.4%)	
	Non-smoker and former smoker	32 (76.2%)	93 (75.6%)	0.940 ^a^
	Active smoker	10 (23.8%)	30 (24.4%)	
	Non-smoker	27 (64.3%)	56 (45.5%)	**0.036 ^a^**
	Active smoker and former smoker	15 (35.7%)	67 (54.5%)	
ICU and invasive mechanical ventilation present		13 (31%)	5 (4.1%)	**<0.001 ^b^**
Death		13 (31%)	12 (9.8%)	**0.001 ^a^**

^a^ Chi-square test; ^b^ Fisher exact test. Bold values were statistically significant.

**Table 3:** Quantitative markers for non-invasive ventilation.

Quantitative Markers	Present (*n* = 42) Median (Q1–Q3)	Absent (*n* = 123) Median (Q1–Q3)	Mann–Whitney U: *p*-Value
Age	72 (65.75–75)	70 (64–78)	0.752
LDH	425 (326.5–695)	336 (240–482)	0.003
PCR	60.1 (9.88–111.33)	15.88 (6.5–57)	0.004
Eosinophile	0 (0–0.01)	0.01 (0–0.08)	0.002
Lymphocytes	0.75 (0.54–1.13)	0.98 (0.72–1.35)	0.017
Leucocytes	8.73 (5.68–11.72)	7.66 (5.92–10.12)	0.429
Thrombocytes	202.5 (157.75–286)	225 (174–300)	0.483
Neutrophiles	7.08 (4.43–9.71)	6.12 (4.32–8.27)	0.346
PLR	257.13 (171.03–452.69)	221.54 (162.1–321.84)	0.109
NLR	10.37 (4.44–14.85)	6.12 (4.07–9.88)	0.020

**Table 4:** Qualitative markers for invasive mechanical ventilation in the ICU.

		Invasive Ventilation		
Qualitative Markers		Present (*n* = 18) *n* (%)	Absent (*n* = 147) *n* (%)	*p*-Value
COVID-19 severity	severe	14 (77.8%)	104 (70.7%)	>0.05 ^b^
	moderate	2 (11.1%)	25 (17%)	
	light	2 (11.1%)	18 (12.2%)	
Consolidation		12 (66.7%)	67 (45.6%)	0.091 ^a^
Number of affected lobes = 0		8 (44.8%)	85 (57.8%)	
Number of affected lobes ≥ 3		8 (44.8%)	18 (12.3%)	**0.002 ^b^**
Cardio-vascular disease present		16 (88.9%)	120 (81.6%)	0.742 ^b^
Arterial hypertension present		14 (77.8%)	122 (83%)	0.526 ^b^
Diabetes present		7 (38.9%)	54 (36.7%)	0.858 ^a^
Renal failure present		4 (22.2%)	18 (12.2%)	0.267 ^b^
Respiratory failure present		6 (33.3%)	49 (33.3%)	>0.05 ^b^
Pre-existing treatment present		6 (33.3%)	112 (76.2%)	**<0.001 ^a^**
O_2_-therapy type at admission	No O_2_	4 (22.2%)	20 (13.6%)	>0.05 ^b^
	NC	0 (0%)	32 (21.8%)	
	SM	4 (22.2%)	18 (12.2%)	
	VM	1 (5.6%)	32 (21.8%)	
	NRM	7 (38.9%)	42 (28.6%)	
	HFOT	2 (11.1%)	3 (2%)	
Smoker status	Non-smoker	13 (72.2%)	70 (47.6%)	>0.05 ^b^
	Former smoker	1 (5.6%)	41 (27.9%)	
	Active smoker	4 (22.2%)	36 (24.5%)	
	Non-smoker and former smoker	14 (77.8%)	111 (75.5%)	>0.05 ^b^
	Active smoker	4 (22.2%)	36 (24.5%)	
	Non-smoker	13 (72.2%)	70 (47.6%)	**0.049 ^a^**
	Active-smoker and former smoker	5 (27.8%)	77 (52.4%)	
Non-invasive ventilation present		13 (72.2%)	29 (19.7%)	**<0.001 ^b^**

^a^ Chi-square test; ^b^ Fisher exact test. Bold values were statistically significant.

**Table 5:** Quantitative markers for invasive mechanical ventilation in the ICU.

Quantitative Markers	Present (*n* = 18) Median (Q1–Q3)	Absent (*n* = 147) Median (Q1–Q3)	Mann–Whitney U: *p*-Value
Age	69 (62.25–74.5)	71 (64–78)	0.430
LDH	425 (326.5–717.25)	349 (244–506)	0.053
PCR	16.44 (7.78–93.03)	20.6 (7.19–70)	0.576
Eosinophile	0 (0–0.01)	0.01 (0–0.07)	0.031
Lymphocytes	0.69 (0.57–0.91)	0.98 (0.71–1.34)	0.012
Leucocytes	8.88 (6.28–14.67)	7.77 (5.84–10.12)	0.234
Thrombocytes	222 (152.25–324)	222 (172–298)	0.724
Neutrophiles	7.86 (5.67–13.06)	6.29 (4.31–8.35)	0.083
PLR	291.89 (212.83–476.66)	232.97 (162.6–354.08)	0.123
NLR	11.73 (6.73–17.2)	6.13 (4.07–10.33)	0.006

**Table 6:** Qualitative markers for death (univariate logistic regression).

		Death		
Qualitative Markers		Present (*n* = 25) *n* (%)	Absent (*n* = 140) *n* (%)	*p*-Value
COVID-19 severity	Severe	21 (84%)	97 (69.3%)	>0.05 ^b^
	moderate			
	light	2 (8%)	25 (17.9%)	
Consolidation		15 (60%)	64 (45.7%)	0.188 ^a^
Number of affected lobes ≥ 3		9 (36%)	17 (12.1%)	**<0.01 ^b^**
Cardio-vascular disease present		23 (92%)	113 (80.7%)	0.255 ^b^
Arterial hypertension present		20 (80%)	116 (82.9%)	0.776 ^b^
Diabetes present		10 (40%)	51 (36.4%)	0.733 ^a^
Renal failure present		7 (28%)	15(10.7%)	**0.048 ^b^**
Respiratory failure present		11 (44%)	44 (31.4%)	0.219 ^a^
Pre-existing treatment present		15 (60%)	103 (73.6%)	0.166 ^a^
O_2_-therapy type at admission	No O_2_	4 (16%)	20 (14.3%)	≥0.05 ^b^
	NC	0 (0%)	32 (22.9%)	
	SM	3 (12%)	19 (13.6%)	
	VM	3 (12%)	30 (21.4%)	
	NRM	12 (48%)	37 (26.4%)	
	HFOT	3 (12%)	2 (1.4%)	
Smoker status	Non-smoker	16 (64%)	67 (47.9%)	0.308 ^a^
	Former smoker	4 (16%)	38 (27.1%)	
	Active smoker	5 (20%)	35 (25%)	
	Non-smoker and former smoker	20 (80%)	105 (75%)	0.591 ^a^
	Active smoker	5 (20%)	35 (25%)	
	Non-smoker	16 (64%)	67 (47.9%)	0.137 ^a^
	Active smoker and former smoker	9 (36%)	73 (52.1%)	
ICU and Invasive mechanical ventilation present		13 (52%)	5 (3.6%)	**<0.001 ^b^**
Non-invasive ventilation present		13 (52%)	29 (20.7%)	**0.001 ^a^**

^a^ Chi-square test; ^b^ Fisher exact test. Bold values were statistically significant.

**Table 7:** Quantitative markers for death.

Quantitative Markers	Present (*n* = 25) Median (Q1–Q3)	Absent (*n* = 140) Median (Q1–Q3)	Mann–Whitney U: *p*-Value
Age	71 (66.5–76.5)	71 (64–77.75)	0.457
LDH	427 (351–737.5)	343.5 (244–503.75)	0.031
PCR	24 (9.18–95.3)	20.25 (6.53–65.88)	0.233
Eosinophile	0 (0–0.03)	0.01 (0–0.06)	0.221
Lymphocytes	0.77 (0.6–0.99)	0.99 (0.68–1.34)	0.036
Leucocytes	8.61 (5.15–11.82)	7.68 (5.88–10.33)	0.757
Thrombocytes	198 (133.5–277)	225 (174–299.5)	0.139
Neutrophiles	6.47 (3.65–8.75)	6.36 (4.5–9.01)	0.849
PLR	232.97 (157.79–418.86)	239.78 (167.85–359.88)	0.794
NLR	8.61 (4.45–12.54)	6.44 (4.03–10.88)	0.304

**Table 8:** Distribution of patients according to their oxygen-ventilation type and death outcome.

Ventilation	ICU and Invasive Mechanical Ventilation						Total
	Mechanical Ventilation		Mechanical Ventilation Total	No Mechanical Ventilation		No Mechanical Ventilation Total	
	Death	Survival		Death	Survival		
Non-invasive ventilation	9	4	13	4	25	29	42
No ventilation	4	1	5	8	110	118	123
**Total**	13	5	18	12	133	147	165

Poor outcome = death (YES) OR ICU + IMV(YES) OR NIV (YES) = 18 + 12 + 25 = 55. Good outcome = 110 (survival with no ventilation OR survival with no invasive mechanical ventilation).

## Data Availability

The data presented in this study are available on request from the corresponding author.
